# A case of eosinophilic granuloma of the skull in an adult man: a case report

**DOI:** 10.1186/1757-1626-2-9144

**Published:** 2009-12-04

**Authors:** Panagiotis V Kitsoulis, Georgios Paraskevas, Aristidis Vrettakos, Aikaterini Marini

**Affiliations:** 1 Department of Anatomy-Histology-Embryology, Medical School, University of Ioannina, Greece; 2 Department of Anatomy, Medical School, Aristotle University, Thessaloniki, Greece

## Abstract

Eosinophilic granuloma is very rare benign bone tumor which presents in more than 90% in children under the age of ten. There is predominance for males. It is usually found at flat and long bones. The skull and vertebral spine is often affected. We report a case of 57 year-old man who gradually developed local pain at his skull and orbit. A soft, movable, palpable and tender mass was found at the left temporal bone. The pain deteriorated after an accidental injury at skull and remained so. The clinical examination revealed no pathological findings. The patient was a doctor who smoked and consumed alcohol daily. He had a history of cardial infraction and psoriatic arthritis. X-rays and CT revealed a round lytic defect at the skull. Its borders were sharp and its size was 1.6 × 1.8 cm. No periostic reaction or bone formation was noted. Scintigraphy depicted a lytic lesion without radionuclide enhancement. Thus we suspected an eosinophilic granuloma. An attempt to excise the tumor failed as it had already eroded the underlying temporal bone. The external meninga was affected but not the internal one. Histological diagnosis with dominance of Langerhans cells set the diagnosis. A second surgery was done and the eosinophilic granuloma was extracted. After eight months the gap was bridged with plastic heterologous transplant. After the curettage the patient received antibiotics and five cycles of radiotherapy. The aesthetic result was excellent. The patient's head has a normal hairy appearance. No tenderness, swelling or recurrence is recorded until now.

Eosinophilic granuloma is of unknown aetiology but uncontrolled proliferation of Langerhans cells, previous inflammations or tumors and autoimmune disorders are suspected. Due to the co-existence of psoriatic arthritis and eosinophilic granuloma to our patient we assume that an autoimmune mechanism is probable.

## Introduction

Eosinophilic granuloma of bones is the mildest and commonest manifestation of Histiocytosis-X malady [1]. It affects male children in more than 90% of the cases. The lesion usually appears as a single one and is sited flat and long bones, the vertebral spine and the skull. Inflammation, autoimmunity and loss of controlled proliferation of Langerhans cells are the assumed aetiologies. This benign tumor is usually asymptomatic or may appear as a palpable, tender mass over the affected bone. Rarely does it cause epidural hematomas, suppression of bone marrow and pathological fractures. When at skull, headache, neurological symptoms, chronic mastoiditis and exopthalmos may be the findings [2]. The diagnosis is based on the depiction of the lesion with roentgenography, CT, MRI, scintigraphy and is set via histological examination after excision or guided biopsy. Eosinophilic granuloma can heal without treatment while passing from childhood to adulthood. If not, the suggested treatment is the surgical curettage of the tumor or local infusion of cortisone. Chemotherapy, radiotherapy and systemic use of cortisone are effective for multiple bone lesions [3].

We present a case of a man at the age of 57 who developed eosinophilic granuloma at the skull without having any previous history of bone tumors but psoriasic arthritis. To our knowledge this is the first reported case of co-existence of the two diseases.

## Case presentation

A 57-year-old man was admitted on March 2006 to our hospital referring acute pain when accidentally injured by a plastic object at head. He mentioned a palpable, soft, immovable and tender mass at the left temporal bone. His left orbit was also painful. He had already used analgesic and anti-inflammatory drugs. No fever, elevated regional temperature nor lymphadenopathy was recorded. The physical and neurological examination had no pathological signs. He did not present numbness, weakness or remarkable tendon reflexes and muscular tone. The mobility of the head was normal. The vision and hearing was not affected.

The patient had a height of 1.78 m and weighted 80 kgrs. He belongs to Arabic nation (born in Lebanon) but he has the Greek ethnicity living in Greece the last 40 years. 31 years ago psoriasic arthritis was diagnosed with the joint of the left elbow mainly affected. He received no therapy for the disease. 10 years ago he presented acute myocardial infarction and undertook bypass. Since then he received drug therapy for hypertension. The patient smoked 2 packs of cigarettes since the age of 25 and consumed 1-2 glasses of alcohol per day. No family history of tumors is known.

Blood examination revealed no pathological findings, neither elevation of leukocytes nor of erythrocyte sedimentation rate. Heart ultrasound revealed no present pathology.

A first attempt to excise the lesion failed as there was an erosion of the temporal bone. Material for histological examination was extracted and we found a domination of histiocytes. No malignancy was suspected. We continued with radiological depiction of the skull.

**Figure 1:**
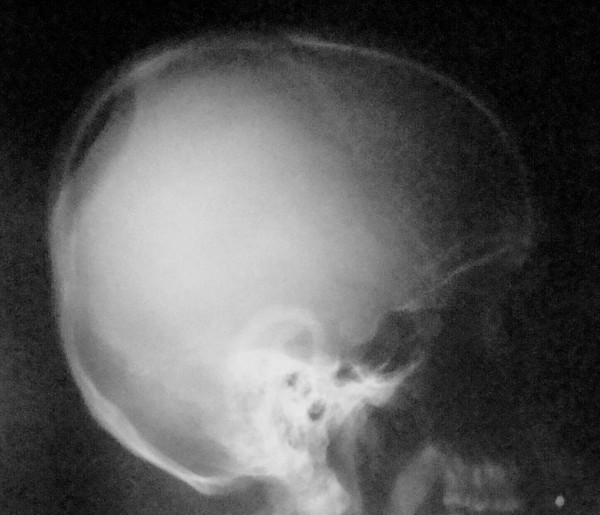
**Plain radiograph of the skull depicting the osteolytic defect on the left temporal bone**.

**Figure 2:**
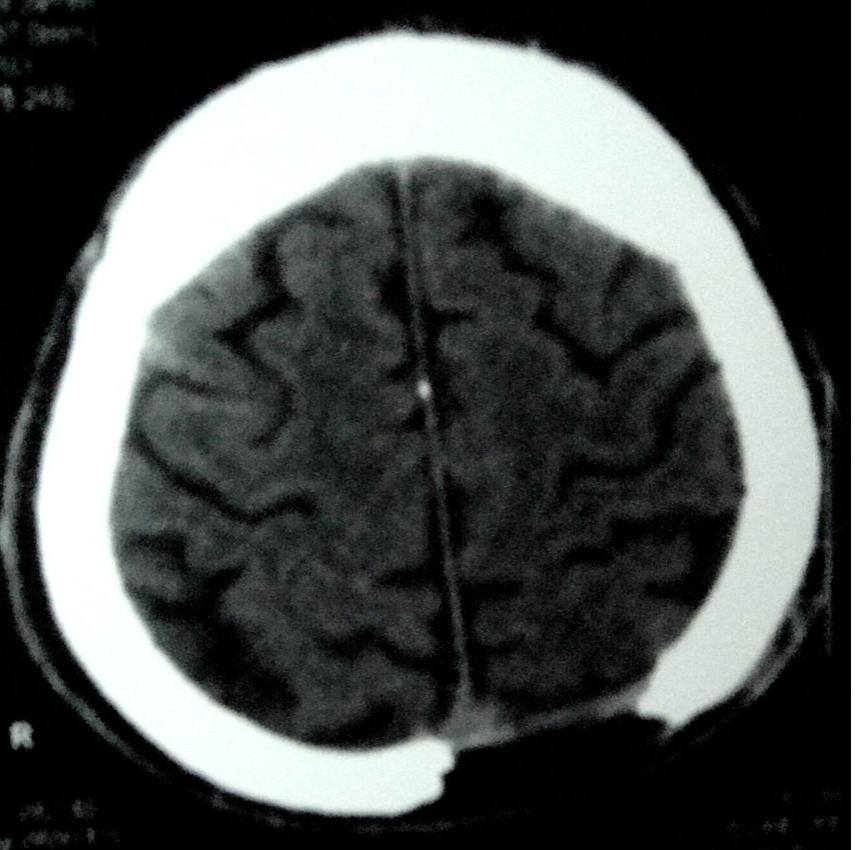
**Computed tomography of the skull**. The tumor appears as a lytic area with sharp borders. Both of brain hemispheres seem intact.

**Figure 3:**
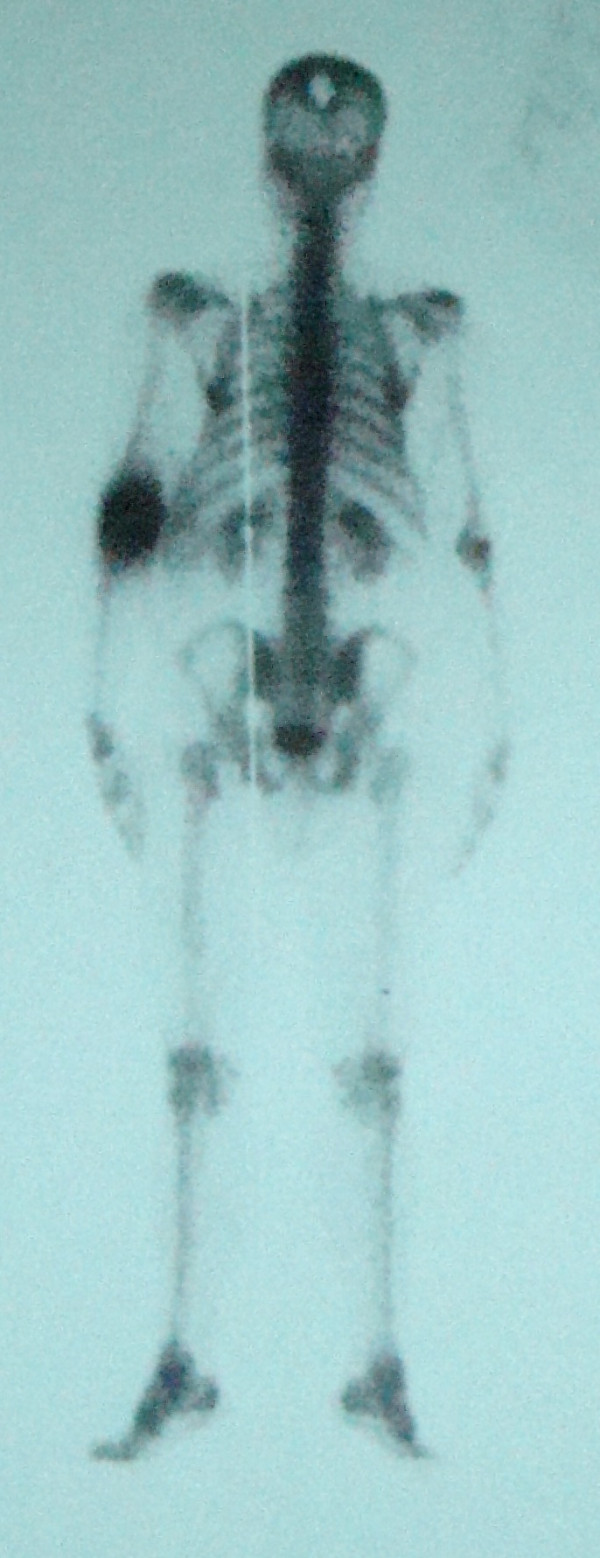
**Bone scintigraphy demonstrating the highly enhanced tumor borders and the left elbow affected by psoriatic arthritis**.

Plain radiographs demonstrated a large oval-shaped osteolytic area in the left temporal bone [figure 1]. Its borders were regular and no bone formation was noted. Its size was about 2 cm. CT verified a lytic lesion at the left temporal bone of the skull [figure 2]. The estimated size was 1.8 × 1.6 cm. The borders were sharp and periosteal reaction was obvious. The meninges were almost exposed to environment and that is the reason why a moderate injury led to intense pain. No fluid was present. Scintigraphy with Tc-99m demonstrated a non absorbent focus in the left temporal bone [figure 3]. Left elbow was also depicted as highly absorbent because of the psoriatic arthritis. No secondary or multiple foci were enhanced.

A radical excision of the lesion on health borders of 2 cm was performed. The tumor was gelatinous and brownish without hematomas. It was perforating the whole diploe and part of the external meninga. We excised a piece of a diameter of 5 cm including the lesion and used a periosteal transplant to bridge the gap. No cranioplasty was done at first time. The patient received vancomycin and cefoxitime for two days. Material was sent for histological examination which showed many Langerhans cells, eosinophils, neutrophils and lymphocytes. No fibroblasts or giant cells were found. The S-100, CD1a and Langering proteins were positive. There were necrotic elements too. Diagnosis of eosinophilic granuloma of the skull was set. The patient was administered five cycles of low-dose post-surgical radiotherapy were administered.

Carnioplasty with a heterologous transplant (Porex) was performed eight months later. The aesthetic result was excellent and there were no complications. On March 2008, a new CT of the brain depicted no further lesion and the aesthetical result was excellent.

## Discussion

Eosinophilic granuloma is one of the rarest bone tumors representing less than 1% of them. In 90% of the reported cases it appears in children under the age of ten. There is a certain predilection to males (2:1) [4]. It usually presents as a monostotic lesion affecting flat and long bones (70%) [5], the skull and the vertebral spine. A unique cell, the Langerhans cell is diagnostic [6]. It contains Birbeck granules whose role is yet unknown. Eosinophils, lymphocytes, fibroblasts and foam cells may be also found but none of them is pathognomonic. The only reliable immunological marker is the OKT6 while the common S-100 protein is usually positive too [7]. Eosinophilic granuloma can be asymptomatic or present as local swelling, pain or tenderness. Depending on the location of the tumor, it may cause neurological symptoms such as numbness, limping, fracture, loosening of teeth, otitis media [8] or exopthalmos. If at skull, then a hematoma after a mild injury is a common finding [9]. No fever or other signs of inflammation have been reported. The blood tests show an elevation of leucocytes and eosinophils in approximately 7% of the cases [10]. Erythrocyte sedimentation rate is over the normal levels. The tumor's material is sterile but there have been reports about the presence of staphylococcus and streptococcus [11].

Radiological depiction of eosinophilic granuloma is necessary as to determine the activity and nature of the tumor. Plain radiograph depicts its size and borders. The cortex of the affected bone may present as thin, eroded or thickened due to new bone formation [12]. Especially at skull there may be one or multiple osteolytic oval defects with regular or irregular borders. CT and MRI demonstrate the exact size and borders of the tumor as well as the situation of the surrounding tissues and a probable hematoma. Eosinophilic granuloma appears with intense signal on CT while on T2 weighted images this signal surpasses that of bone marrow. The aforementioned methods are excellent for imaging the spinal cord [13]. Radionuclide bone scan with technetium, gallium or thallium reveal an enhancing mass and easily detect other foci or recurrence points. Ultrasound is only used for guided biopsies. Diagnosis is set by histological examination.

The aetiology of eosinophilic granuloma remains unknown but recently there have been some assumptions. The prevailing ones are inflammatory processes, autoimmune disorders and an out of control proliferation of Langerhans cell [14]. It is now known that the pathognomonic cell, the Langerhans cell, excretes IL-1 and PG-E2 as to damage surrounding tissues. Though not a proven affiliation, our patient already had an autoimmune disease (psoriasis) before the appearance of eosinophilic granuloma. To date only two studies report the co-existence of psoriasic arthropathy with eosinophilic fasciitis or folliculitis [15,16]. Thus, we make the tentative hypothesis that the autoimmunity and atopic diathesis is the common aetiological basis of the two diseases.

Eosinophilic granuloma does not lead to malignant transformation. If it expands elsewhere but bones then is called Hand-Schuller-Christian disease and may manifest as diabetes insipidus, cerebellar, hypothalamic and with other central nervous system symptoms. The prognosis depends on the age of diagnosis and the number of foci.

This tumor may heal without treatment but there is a chance of relapsing during the first year after diagnosis. Thus, even after surgical curettage a careful follow-up must be conducted. If a secondary lesion appears then chemotherapy or further excision has to be done [17].

## Conclusion

This is one of the rarest cases of eosinophilic granuloma of the skull developing in an adult patient. This is the first reported case of co-existence of eosinophilic granuloma with psoriatic arthritis. It is important to include eosinophilic granuloma in the differential diagnosis of bone lesions in adult subjects because of the possible expansion of the disease if untreated. We suggest surgical curettage and plastic bone transplant at second time and radiotherapy as principal treatment. A follow-up of a year is necessary for probable recurrence.

## Abbreviations

CT: computed tomography; IL: interleukin; MRI: magnetic resonance imaging; PG: prostaglandin.

## Consent

Written informed consent was obtained from the patient for the publication of this case report and accompanying images. A copy of the written consent is available for review by the Editor-in-Chief of this journal.

## Competing interests

The authors declare that they have no competing interests.

## Authors' contributions

All authors contributed equally to this work. PK concentrated the patient's data, histological examinations, history and radiological depictions. PK and AV interpreted the findings and contributed in writing the manuscript. AM and GP analyzed the patient data, reviewed the current literature and contributed in writing the manuscript. All authors read and approved the final manuscript.
